# Identification of biomarkers predictive of metastasis development in early-stage colorectal cancer using network-based regularization

**DOI:** 10.1186/s12859-022-05104-z

**Published:** 2023-01-16

**Authors:** Carolina Peixoto, Marta B. Lopes, Marta Martins, Sandra Casimiro, Daniel Sobral, Ana Rita Grosso, Catarina Abreu, Daniela Macedo, Ana Lúcia Costa, Helena Pais, Cecília Alvim, André Mansinho, Pedro Filipe, Pedro Marques da Costa, Afonso Fernandes, Paula Borralho, Cristina Ferreira, João Malaquias, António Quintela, Shannon Kaplan, Mahdi Golkaram, Michael Salmans, Nafeesa Khan, Raakhee Vijayaraghavan, Shile Zhang, Traci Pawlowski, Jim Godsey, Alex So, Li Liu, Luís Costa, Susana Vinga

**Affiliations:** 1 INESC-ID, Instituto Superior Técnico, Universidade de Lisboa, Rua Alves Redol 9, 1000-029 Lisbon, Portugal; 2 NOVA Laboratory for Computer Science and Informatics (NOVA LINCS), NOVA School of Science and Technology, 2829-516 Caparica, Portugal; 3 Center for Mathematics and Applications (NOVA MATH), NOVA School of Science and Technology (FCT NOVA), 2829-516 Caparica, Portugal; 4 Instituto de Medicina Molecular - João Lobo Antunes, Faculdade de Medicina de Lisboa, Avenida Professor Egas Moniz, 1649-028 Lisbon, Portugal; 5 Oncology Division, Hospital de Santa Maria, Centro Hospitalar Lisboa Norte, Lisbon, Portugal; 6 Associate Laboratory i4HB - Institute for Health and Bioeconomy, NOVA School of Science and Technology, Universidade NOVA de Lisboa, 2829-516 Caparica, Portugal; 7 UCIBIO - Applied Molecular Biosciences Unit, Department of Life Sciences, NOVA School of Science and Technology, Universidade NOVA de Lisboa, 2829-516 Caparica, Portugal; 8 Illumina Inc., 5200 Illumina Way, San Diego, CA 92122 USA; 9 IDMEC, Instituto Superior Técnico, Universidade de Lisboa, Av. Rovisco Pais, 1, 1049-001 Lisbon, Portugal

**Keywords:** Colorectal cancer, Classification, Biomarker selection, Regularization, iTwiner

## Abstract

Colorectal cancer (CRC) is the third most common cancer and the second most deathly worldwide. It is a very heterogeneous disease that can develop via distinct pathways where metastasis is the primary cause of death. Therefore, it is crucial to understand the molecular mechanisms underlying metastasis. RNA-sequencing is an essential tool used for studying the transcriptional landscape. However, the high-dimensionality of gene expression data makes selecting novel metastatic biomarkers problematic. To distinguish early-stage CRC patients at risk of developing metastasis from those that are not, three types of binary classification approaches were used: (1) classification methods (decision trees, linear and radial kernel support vector machines, logistic regression, and random forest) using differentially expressed genes (DEGs) as input features; (2) regularized logistic regression based on the Elastic Net penalty and the proposed iTwiner—a network-based regularizer accounting for gene correlation information; and (3) classification methods based on the genes pre-selected using regularized logistic regression. Classifiers using the DEGs as features showed similar results, with random forest showing the highest accuracy. Using regularized logistic regression on the full dataset yielded no improvement in the methods’ accuracy. Further classification using the pre-selected genes found by different penalty factors, instead of the DEGs, significantly improved the accuracy of the binary classifiers. Moreover, the use of network-based correlation information (iTwiner) for gene selection produced the best classification results and the identification of more stable and robust gene sets. Some are known to be tumor suppressor genes (*OPCML-IT2*), to be related to resistance to cancer therapies (*RAC1P3*), or to be involved in several cancer processes such as genome stability (*XRCC6P2*), tumor growth and metastasis (*MIR602*) and regulation of gene transcription (*NME2P2*). We show that the classification of CRC patients based on pre-selected features by regularized logistic regression is a valuable alternative to using DEGs, significantly increasing the models’ predictive performance. Moreover, the use of correlation-based penalization for biomarker selection stands as a promising strategy for predicting patients’ groups based on RNA-seq data.

**Supplementary Information:**

The online version contains supplementary material available at 10.1186/s12859-022-05104-z.

## Introduction

Colorectal cancer (CRC) is one of the leading causes of cancer-related deaths worldwide. In 2018, it was the third most common cancer, with around 1.8 million new cases and the second most deathly cancer with almost 900 thousand deaths (9% of all cancer-related deaths) [1]. CRC begins as a benign adenomatous polyp, which develops into an advanced adenoma with high grade dysplasia and then progresses to invasive cancer. Invasive cancers that are confined within the wall of the colon (stages I and II) are curable. However, if untreated, they may spread to regional lymph nodes (stage III) or later metastasize to distant sites (stage IV) [2].

CRC is a very heterogeneous disease that can develop via distinct pathways involving different combinations of genetic and epigenetic changes [3]. These genetic differences between patients may lead to differences in susceptibility where cancers deriving from the same tissue may be stratified into disease subtypes [4]. Genetic and epigenetic heterogeneity poses a problem for the diagnosis and therapy of cancer. For example, it can lead to incorrect treatment decisions. CRC has three main types known, divided by their origin and expression: sporadic form (60%–80% of the cases), family type (20%–40%) and hereditary type [5]. Sporadic CRC may appear in individuals who carry no mutation that makes them susceptible to developing this type of cancer. Regarding the family type, no gene has been found to be related to the disease. However, there is a higher chance of developing this tumor when family members have suffered from sporadic colon cancer. In these cases, environmental factors play a critical role. Hereditary type may be divided into two subtypes whether patients show adenomatous polyps - familial adenomatous polyposis (FAP), or not - hereditary nonpolyposis colorectal cancer [5].

Metastasis is the major cause of death in CRC patients, and approximately 20% of the patients already have metastases at diagnosis [6]. In this context, it is vital to diagnose CRC at an early-stage and accurately identify patients likely to progress to metastasis in order to improve CRC patients outcomes. Tumor surgical removal is the treatment of choice for early localized CRC disease (stage II-III) [7]. 50% of stage III patients are cured by surgery, whereas 20% of patients will survive due to the addition of adjuvant chemotherapy and 30% will relapse in 2-3 years [8]. Altogether, only 20% of stage III patients benefit from chemotherapy, exposing 80% of patients to unnecessary toxicity [9]. Therefore, one of the main challenges is to identify those stage II-III CRC patients where adjuvant chemotherapy is crucial to improve their outcomes.

Many studies try to understand tumor biology and mechanisms that lead to metastasis; notwithstanding, the identification of the factors influencing metastatic tumor cells, especially in colorectal cancer, remains poor [10]. Consequently, over the years, there was an increase in molecular profiling of tumors using next-generation sequencing (NGS), such as RNA sequencing (RNA-seq), which constitutes an important tool widely used in cancer research for studying the transcriptional landscape and molecular pathways [11].

Supervised learning comes as a natural choice for helping in the classification of patients into metastatic and non-metastatic, based on NGS data. Some of the widely used classifiers applied to RNA-seq data are Logistic Regression (LR), Decision Tree (DT), Random Forest (RF), and Support Vector Machine (SVM) [12–14]. However, despite the invaluable information provided by NGS, the intrinsic high dimensionality of gene expression data may compromise the classification learning task and severally hamper an accurate selection of biomarkers. Therefore, feature selection plays a pivotal role in the selection of informative genes preceding the classification of RNA-Seq data for disease prediction and diagnosis, to enhance accuracy in disease classification [15]. Furthermore, ranking of the features according to their relevance to the classification problem and further selection of the best ones can improve the performance of the prediction model [13].

Another common way to address the data high-dimensionality challenge is to use classification algorithms that control the model’s complexity through regularization [16, 17]. One option is to regularize the log-likelihood function of the LR model. Two of the most commonly used penalties are the lasso ($\ell _1$-norm) and the ridge ($\ell _2$-norm) [14] regularizers, whose combination leads to the Elastic Net [18]. Network analysis has also shown enormous potential in precision medicine, helping to identify key biomarkers and therapeutic targets in cancer [19]. Several studies used network-based regularizers to improve model accuracy and interpretability. Prior network knowledge may be based on protein-protein interactions [20], or from the correlation matrix of the gene expression values [21, 22].

In sum, several studies demonstrated that using supervised learning methods in microarray gene expression data [23] is a very promising technique and that the integration of gene expression profiles with network information may help to identify markers correlated with metastasis [24]. Also, in the context of colorectal cancer, some classifiers developed to investigate metastasis were based only on clinical data (e.g., sex, age at diagnosis, histological subtype, stage, primary site) [25]. Therefore, there is still an urgent need of methods for the identification of factors influencing metastatic tumor cells, especially in colorectal cancer.

In this work, we try to find a set of biomarkers that may predict the risk of metastasis using transcriptomic data from a cohort of CRC patients followed at the Hospital de Santa Maria (Lisbon), one of the largest hospitals in Portugal.

To achieve this goal, we applied and tested different classification methods using transcriptomic data, and proposed a new combined model that showed higher classification accuracy compared to its model counterparts. Altogether, we proposed a new pipeline for the selection of putative biomarkers based on patients’ gene correlation matrices.

## Materials and methods

**Fig. 1:**
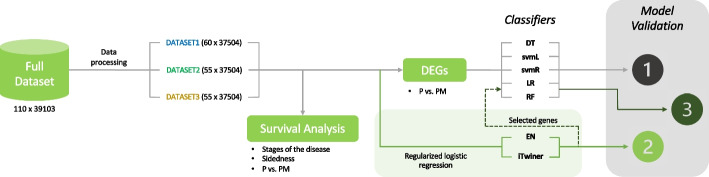
Methodological procedure of the work presented here. The full dataset was divided into three smaller datasets. Survival analysis was performed to each dataset to evaluate how stages of the disease (II vs. III), sidedness of primary tumor site in colon (Right vs. Left), and class (P—primary patients that do not metastasize vs. PM—primary patients that metastasize) are related to risk of death. Afterwards, three different approaches to classify early-stage patients that metastasize were used: (1) Classifiers without regularization (DT – decision trees, svmL—linear support vector machine, svmR—radial support vector machine, LR—logistic regression and RF—random forest) applied to subset of genes that were found differentially expressed between two groups (P vs. PM); (2) Regularized logistic regression performed on the full dataset using two different penalization factors (EN—elastic net, and iTwiner); (3) Classifiers applied to genes pre-selected by regularized logistic regression. Model performance was compared using different types of measures (e.g., accuracy and misclassifications)

To identify important genes involved in the CRC metastasis process, several classification methods applied to RNA-seq data were tested. The pipeline of this study is represented in Fig. 1. All the methods were implemented in the R statistical software [26] and the corresponding code is available at https://github.com/sysbiomed/iTwiner.git.

### Datasets

Primary tumor samples from patients diagnosed with CRC disease from June 2010 to October 2017 were collected as part of a prospective biobanking project approved by the Ethical Committee of Hospital de Santa Maria, all procedures were performed in accordance with relevant guidelines. Patients were followed at the Oncology Division of Hospital Santa Maria, Lisbon, and were treated as per institutional clinical practice in accordance with international guidelines, namely ESMO and NCCN guidelines. Cases were staged according to The American Joint Committee on Cancer (AJCC) staging system, 8th edition, and patients had not received neoadjuvant chemo or radiotherapy prior to sample collection. Whole transcriptome sequencing (WTS) was performed by Illumina Inc.

The dataset used in this study comprises 110 samples from early-stage (II and III) CRC patients with both clinical and transcriptomic (RNA-seq) data. This was obtained from two different cohorts of CRC patients from Hospital Santa Maria (Lisbon, Portugal): Cohort 1: Cohort described in [27] containing 111 samples, available under accession number EGAS00001005276 (European Genome-Phenome Archive). This cohort has 26 samples from primary stage II-III colorectal tumors that did not metastasize, 34 primary stage II-III colorectal tumors that metastasize in three years of follow up, 12 adjacent normal colonic mucosa, and 39 metastasis of CRC patients. From this cohort, only the primary colorectal tumors samples were used ($n_{T1}=45$), from early-stage CRC that metastasize ($n_{PM1}=19$), and did not metastasize ($n_{P1}=26$). Cohort 2: Cohort described in [28] containing 114 samples, already available in NCBI Database under accession number PRJNA689313. We used $n_{T2}=65$ samples that correspond to early-stage CRC that metastasize ($n_{PM2}=11$) and early-stage CRC that did not metastasize ($n_{P2}=54$).

The clinical dataset descriptive statistics are summarized in Table 1. The sex (Female or Male), tissue of cancer primary site (Colon or Rectum), stage of the disease (II or III), sidedness of primary site (Right or Left side of the colon), and age variables were selected for further analysis. For the classification methods, two groups of interest were selected, early-stage (II-III) patients that do not metastasize (P, $n_{P1}+n_{P2}=80$) and early-stage patients that metastasize (PM, $n_{PM1}+n_{PM2}=30$) during the follow-up time period. Given the resulting imbalanced groups and the problems in classification that were obtained due to class imbalance, an undersampling strategy was taken for model training by splitting the initial dataset into three different smaller datasets, i.e., DATASET1 ($n = 60$), DATASET2 ($n = 55$), and DATASET3 ($n = 55$). For each dataset, PM patients were the same ($n = 30$) and P patients were randomly divided into three groups ($n_1 = 30$, $n_2 = 25$, $n_3 = 25$). With this strategy, we exploit all the data collected while keeping class balance in each classification procedure. Other data partitions may be tested using the available code.

The original gene expression dataset was comprised of 39,103 variables (genes). After excluding the genes with a constant expression (standard deviation of zero), a dataset with 37,504 variables (genes) was obtained.

A preliminary study of the datasets was performed to verify the statistical significance of the differences between factor variables across the P and PM groups of patients using the Fisher’s Exact test, namely to the variables sex, tissue type, stage of the disease, and sidedness. For the age variable, a t-test was used to compare the mean between the two groups. Subsequent survival analysis was performed for each dataset used, with the main goal of studying the time until an event of interest, i.e., death, occurs [29]. Here, we compared the differences in survival between several groups of interest – namely, stages of the disease (II vs. III), sidedness (Right vs. Left side of the colon), and class (P vs. PM) – using the log-rank test [30].

Finally, differential gene expression analysis was performed to identify genes differentially expressed between the two patient groups (P and PM). To perform this analysis, the edgeR R software package was used, employing an FDR (false discovery rate) cut-off of 0.05 to identify differentially expressed genes (DEGs). These genes were further used for classification.

**Table 1:** Distribution of the patients of each dataset (D1 - DATASET1, D2 - DATASET2, D3 - DATASET3) used regarding sex (Female, Male), tissue type (Colon, Rectum), stage of the disease (II, III), sidedness (Right, Left) and age; * *p*-value comparing P and PM class groups using the Fisher exact test

	Total			*P*			PM (30)	*p*-value*		
	D1 (60)	D2 (55)	D3 (55)	D1 (30)	D2 (25)	D3 (25)	–	D1	D2	D3
**Sex**										
Female	37	30	30	20	13	13	17			
Male	23	25	25	10	12	12	13	0.60	0.79	0.79
**Tissue**										
Colon	53	49	47	28	24	22	25			
Rectum	7	6	8	2	1	3	5	0.42	0.20	0.72
**Stage**										
II	32	24	24	22	14	14	10			
III	28	31	31	8	11	11	20	0.004	0.11	0.11
**Sidedness**										
Right	23	23	24	14	14	15	9			
Left	26	21	17	13	8	5	13	0.32	0.12	0.04
**Age**	68.55	67.77	69.5	68.29	66.52	70.36	68.77	0.90	0.53	0.64

Regarding age, a* t*-test was used to compare the mean between groups

### Classification methods

Classification is a supervised learning method, where the model learns from a set of predefined samples with given class labels (training dataset). The knowledge inferred from this is applied to classify unknown samples (a test dataset) accordingly [13].

In this work, three binary classification approaches were used to distinguish early-stage CRC patients that metastasize from those that did not: 1) classification methods based on a subset of relevant genes (DEGs), 2) classification via regularized logistic regression with embedded feature selection applied to the full dataset, and 3) all classifiers based on the relevant features identified by regularized logistic regression (Fig. 1).

#### Binary classification

Regarding binary classification, five different classifiers were tested: decision trees, support vector machines (linear and radial), logistic regression, and random forest. One of the limitations of these methods emerges when using high-dimensional data. Since a high number of features may lead to problems in classification analysis, a smaller subset containing only genes found to be differentially expressed between the groups P and PM was used. The difference in the expression level of genes is found useful in classification in order to identify disease biomarkers [13]. Decision trees (DT) are one of the most used classifiers. The tree complexity, measured by the number of nodes and number of features used, has a crucial effect on its accuracy  [31]. Certain parameters for tree construction were fixed as explained in the section “Method evaluation and comparison”. SVMs have been successfully applied to a wide variety of biological applications, such as the classification of microarray gene expression profiles. Here we tested both linear (svmL) and radial (svmR) kernel functions [32]. Logistic regression allows the analysis of binary outcomes using a logistic function [33]. This method will be explained in more detail below. Finally, Random forest (RF) is an ensemble learning method for classification, operating by constructing a multitude of decision trees [34] to get a more accurate and stable prediction. These classification procedures were performed using the R software caret package.

#### Regularized logistic regression

Another approach that has been widely used for classification problems in cancer is logistic regression [35, 36]. This method is used for modeling a binary response variable [37]. In this specific case, we investigated how metastasis may be predicted using gene expression levels from early-stage CRC patients.

The logistic regression model estimates the probability of belonging to a given class ($Y_i=1$) by:

1 $$\begin{aligned} P({Y}_i=1|{{\textbf {X}}}_i,{\varvec{\beta }})=\frac{\exp ({{\textbf {X}}}_i^T {\varvec{\beta }})}{1+\exp ({{\textbf {X}}}_i^T {\varvec{\beta }})}, \end{aligned}$$

where ${{\textbf {X}}}_{i}, i = 1,\ldots ,n$, is the vector of the *p* covariates (gene expression values) of the *i*-th patient, and ${{\varvec{\beta }}} = (\beta _{1}, \beta _{2}, \dots ,\beta _{p})$ are the corresponding regression coefficients.

The parameters ${{\varvec{\beta }}}$ of the logistic model are estimated by maximizing the log-likelihood function, given by

2 $$\begin{aligned} l({\varvec{\beta }})&= \sum _{i=1}^{n} \Big \{y_i \log P (Y_i=1|{{\textbf {X}}}_i,{{\varvec{\beta }}}) + (1 - y_i) \log [1 - P (Y_i=1|{{\textbf {X}}}_i,{{\varvec{\beta }}})]\Big \}, \end{aligned}$$

where the binary variable $y_i$ indicates to which group observation *i* belongs to, either a patient known to have metastasized in the future (group PM, $y_i=0$) or to a patient whose tumor did not metastasize (group P, $y_i=1$).

One of the most used techniques to handle high-dimensional gene expression data is regularization [38]. The most common regularizer is the Elastic Net ([18]), which combines the $\ell _1$-norm and the squared $\ell _2$-norm of the parameters:

3 $$\begin{aligned} F({\varvec{\beta }}) = \lambda \Big \{ \alpha \left\Vert {\varvec{\beta }} \right\Vert _1 + (1-\alpha ) \left\Vert {\varvec{\beta }} \right\Vert _2^2 \Big \}, \end{aligned}$$

where $0 \le \alpha \le 1$. When $\alpha =1$, the least absolute shrinkage and selection operator (Lasso) is obtained, whereas $\alpha =0$ corresponds to the Ridge regression. Lasso may set coefficients to zero, resulting in a sparse model with fewer coefficients. Ridge regression, on the other hand, is a continuous shrinkage method that minimizes the residual sum of squares, keeping all the predictors in the model [39]. The parameter $\lambda$ that controls the penalizing weight is usually chosen with cross-validation.

Incorporating network-based regularizers in classifiers may improve model interpretability leading to parameter estimation towards meaningful biological solutions. This network information may be obtained from the data correlation itself. For example, Twiner was recently proposed as a regularizer based on pairwise correlations between the features in two distinct groups A and B [21]. This method allows the selection of similarly correlated genes in two groups (e.g., in two given diseases). Here we propose a variant of Twiner, the iTwiner, in which the more different a gene’s correlation pattern is between two groups (metastatic and non-metastatic), the less penalized will be in the regularization term of logistic regression.

Given two correlation matrices for *A* and *B*, ${\Sigma _A = [ {\varvec{\sigma }}_1^A,...,{\varvec{\sigma }}_p^A}]$ and ${\Sigma _B = [ {\varvec{\sigma }}_1^B,...,{\varvec{\sigma }}_p^B}]$, respectively, where each column ${\varvec{\sigma }}_j \in \mathbb {R}^p$ represents the correlation of each feature $j=1,\ldots ,p$ with the remaining ones, the dissimilarity measure $d_j(A,B)$ of feature *j* between *A* and *B* is given by the angle of the corresponding vectors

4 $$\begin{aligned} d_j(A,B) = \arccos { \frac{<{\varvec{\sigma }}_j^A,{\varvec{\sigma }}_j^B>}{\Vert {\varvec{\sigma }}_j^A \Vert \cdot \Vert {\varvec{\sigma }}_j^B \Vert } }, \quad j=1,\ldots , p. \end{aligned}$$

The regularizer is constructed using these distances, to promote the selection of genes whose correlation patterns are more distant between *A* and *B*. The penalty term is given by

5 $$\begin{aligned} F({\varvec{\beta }}) = \lambda \Big \{ \alpha \Vert \textbf{q} \circ {\varvec{\beta }} \Vert _1 + (1-\alpha ) \Vert \textbf{q} \circ {\varvec{\beta }} \Vert ^2_2 \Big \}, \end{aligned}$$

where vector $\textbf{q}=(w_1^{-1},\ldots ,w_j^{-1},\ldots ,w_p^{-1})$ represents the inverse of the normalized distances $w_j = d_j(A,B) / \max _k d_k(A,B)$.

The iTwiner adapts the former regularization in order to penalize, now in an inverse way, the gene expression correlation similarities between the two groups (P vs. PM). The main rationale, in this context, is to select biomarker signatures that indeed reflect the different correlation patterns between the metastatic vs. non-metastatic early-stage CRC patients.

### Method evaluation and comparison

In this work we tested three different approaches to find the best CRC metastasis classifier: 1) Classifiers based on DEGs; 2) Regularized logistic regression applied to the full dataset; 3) Classifiers based on genes pre-selected from regularization (instead of DEGs).

As explained above, the original dataset was split into three smaller datasets due to the existing class imbalance. For each dataset used, samples were randomly divided into a training set (for model construction) and a test set (for model evaluation), comprising 70% and 30% of the data, respectively. To obtain statistically reliable predictive measurements, 10-fold cross-validation was performed on the training set to optimize the $\lambda$ parameter in regularized logistic regression. Regarding decision trees, the minimum number of observations that must exist in a node in order for a split to be attempted and the minimum number of observations in the final node were fixed (minsplit = 4; minbucket = minsplit/3, respectively). After testing manually some values, these were the ones that gave the best estimated tree. Also, a 10-fold cross-validation was used across all runs to tune maxdepth and estimate the best tree, guaranteeing models’ comparison. This estimation procedure and hyper-parameter optimization was performed using the R software package rpart. For support vector machine, random forest and logistic regression classifiers, the train function from caret package was used to perform hyper-parameter optimization from a training set using the default 10-fold cross-validation. To mitigate the variability of these procedures, train and test sets were randomly generated 100 times, keeping the same fixed split (70%-30%).

For the EN model the parameter that controls sparsity was set to $\alpha = 0.2$ and for iTwiner $\alpha = 0.05$, which selected an adequate number of variables to be further analyzed and interpreted. Notwithstanding, different $\alpha$ parameters may be tested to select different gene set sizes, using the code made available.

To evaluate the models’ performance, depending on the class predicted by the classifier and the true class of the patient (non-metastatic - P or metastatic - PM), four different results can be obtained: True positive (TP) - patient predicted as positive (non-metastatic) and the patient was non-metastatic; False positive (FP) - patient predicted as positive (non-metastatic) but the patient did metastasize; True negative (TN) - patient predicted as negative (metastatic) and the patient metastasized; False negative (FN) - patient predicted as negative (metastatic) but the patient did not metastasize. Using these results, the following measures on the test set were used as indicators of the performance of the classifiers: Accuracy (fraction of correct predictions - Acc), number of misclassifications (Miscl), Sensitivity (fraction of actual positive cases), Specificity (fraction of actual negative cases) and AUC (area under the ROC curve). The median values of all performance indexes obtained for train and test sets across the 100 runs were used for comparison.

To perform the analysis described above, glmnet [40] package was used in R statistical software. The ${{\textbf {q}}}$ vector was introduced as a penalty factor in the glmnet function.

## Results and discussion

Different gene expression profiles are expected in early-stage patients that will metastasize compared to non-metastatic patients, as a consequence of molecular, biochemical, and genetic variations that make metastatic cells able to migrate from the primary tumor to other body sites [41]. In this work, several classification and feature selection strategies based on RNA-seq data were evaluated to distinguish early-stage (II-III) CRC patients that metastasize from those that do not, and to find a subset of genes that may be predictive of CRC metastasis.

### Exploratory analysis

The data used to perform this analysis is described in Table 1. As explained before, the full dataset was divided into three. These were analyzed individually and patients were divided into several groups regarding important clinical factors such as sex (Female and Male), tissue (Colon and Rectum), stages of the disease (II and III), sidedness (Right, Left), and age. The statistical significance of the differences between groups P and PM for each clinical factor can be found in Table 1. Most clinical factors yielded no significance in the differences between groups. This is an important step to guarantee that further differences found between P and PM groups are related to gene expression data and not to possible clinical confounding factors.

**Fig. 2:**
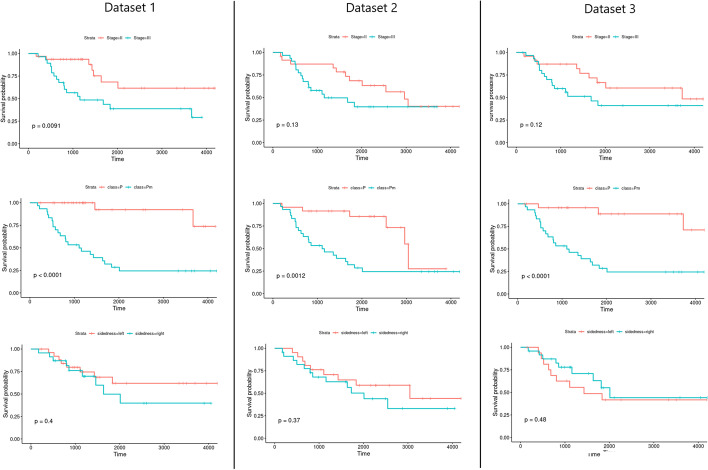
Survival curves for each dataset used, regarding different stages—II vs. III (top line), class—P vs. PM (mid line) and sidedness—Right vs. Left (bottom line)

Afterward, to assess if there were differences in the survival probability regarding clinical factors, survival analysis was performed (Fig. 2) for each dataset used, and the significance of the differences was determined via the log-rank test. As expected, stage III of the disease (more advanced stage), was related to a higher risk of death compared to stage II. This was observed in all datasets, however, only DATASET1 had significant results (*p* value = 0.0091). Also, PM patients showed worst survival probability when compared to P patients, significant in all datasets (*p* value < 0.001). Finally, regarding sidedness, no statistically significant results were found. However, for DATASET1 and DATASET2, there was a tendency for the right side to be related with worst survival probability, as shown in the literature [42].

**Fig. 3:**
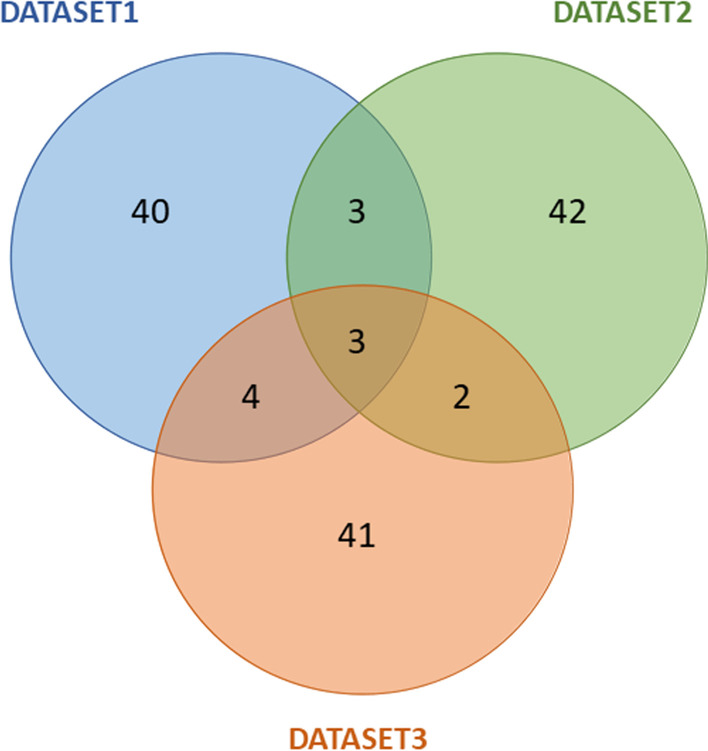
Venn’s diagram comparing fifty DEGs found in each dataset, that exhibit the lowest *p*-values between the P and PM groups of patients

Differential gene expression analysis was performed in all datasets to find differential expressed genes (DEGs) between P and PM patient groups. In DATASET1, a total of 9533 DEGs were found. Among those, 1589 were up-regulated and 7944 down-regulated in PM patients. In DATASET2, 1840 DEGs were found, 835 up-regulated and 1005 down-regulated in PM. Finally, 138 DEGs were found in DATASET3, 39 up-regulated and 99 down-regulated. Given the high number of DEGs found in each dataset, a smaller gene set containing only the fifty DEGs that exhibited the lowest *p*-values between the two tissues was created, for ease of model building and interpretation. The list of ranked genes can be found in Table 2. To compare if there were DEGs found in common between datasets, a Venn diagram was constructed (Fig. 3).

The DEGs found in common between datasets are represented in Table 3, where log fold change (LogFC) is also shown. Negative values refer to down-regulated genes and positive values to up-regulated genes in PM patients. As we can see, twelve genes were considered DEGs in at least two datasets between tissues of early-stage metastatic patients and non-metastatic, with 3 DEGs in common between all of the datasets tested, *GBP4, IDO1, IGHV4-34*. Interestingly, all these three genes have important implications in immune regulation, highly relevant for cancer progression [43–45]. Several of the other genes identified have previously been involved in cancer cells migration, invasion and metastasis such as *LRP4, LGR6, APOL1* and *CXCL11* [46–49].

**Table 2:** List of the top fifty genes with lowest *p*-value found between P vs. PM, regarding different gene expression analysis, subsequently used for classification analysis in each dataset tested

**DATASET1**	**DATASET2**	**DATASET3**
*LRP4*	*CHGA*	*SPOCK2*
*AXIN2*	*TPH1*	*LGR6*
*MIR3197*	*VWA5B2*	*ORM1*
*TFF2*	*STXBP5L*	*H2BS1*
*RNU7-77P*	*EGFR*	*MTRNR2L12*
*GBP4*	*IDO1*	*NELL2*
*RNU6-83P*	*PEG3*	*CXCL11*
*IDO1*	*MIR3978*	*IDO1*
*RNU6-769P*	*NLRP2*	*SLCO1B1*
*LGR6*	*PTPRN*	*SLCO1B7*
*RNU6-580P*	*RNU6-1010P*	*CD8A*
*RNU6-196P*	*RIMS2*	*CNN1*
*S100A12*	*CPLX2*	*HTR1D*
*RNU6-1082P*	*LRP4*	*ACTG2*
*MIR567*	*MIR5003*	*SLC38A11*
*SNORD66*	*NELL2*	*ABCA12*
*CD8B*	*IGLV10-54*	*HLA-DRB5*
*KIT*	*RNU6-196P*	*ASB4*
*MIR6895*	*IGHV4-34*	*GBP4*
*WIPF3*	*ADGRV1*	*CALB1*
*MIR559*	*RET*	*APOL1*
*RNU6-1176P*	*CXCL11*	*FBXO39*
*BIRC3*	*FMN2*	*DES*
*DUOX2*	*GBP4*	*CRYAB*
*MIR28*	*SCN3A*	*IGHV4-34*
*ZPR1*	*CYP3A7*	*ITGAE*
*RNU6-1111P*	*USH2A*	*MIR155HG*
*RNU6-593P*	*SORCS2*	*TNNT2*
*SLC28A2*	*MUC16*	*PLAAT4*
*RNU6-912P*	*RNU6-1208P*	*OSR2*
*RNU2-69P*	*RNA5SP241*	*IGHD*
*RNU6-310P*	*MX1*	*DTHD1*
*SNORA36B*	*MAP6*	*GZMH*
*RNU6-223P*	*SHISA2*	*LINC02323*
*RNU2-24P*	*RNU6-122P*	*MGP*
*ATAD3C*	*MAP2*	*SLFN12L*
*SCARNA23*	*RNU6-677P*	*CD8B*
*MIR4639*	*CCDC158*	*SNORD116-14*
*RNU5B-1*	*TTLL6*	*KLK7*
*RNA5SP179*	*CDHR3*	*FABP3*
*RNU6-807P*	*SYT16*	*CX3CL1*
*RNU7-73P*	*KIF5C*	*TNNC2*
*TAP1*	*MIR7-3HG*	*MYL9*
*IGHV4-34*	*WIPF3*	*TIGIT*
*CD8A*	*CMPK2*	*AIM2*
*S100A8*	*RUNDC3A*	*THEMIS*
*RNU7-19P*	*AIRE*	*IFI44L*
*RNU7-70P*	*NCAM1*	*CD7*
*RNU6-767P*	*MIR552*	*LINC02446*
*APOL1*	*CAMK2B*	*WARS1*

**Table 3:** DEGs found in common at least in two datasets used with fold change regarding primary patients that will metastasize (PM). logFC - log fold change; Multiple testing correction is performed by applying the Benjamini-Hochberg method on the *p*-values, to control the false discovery rate (FDR)

	DATASET1		DATASET2		DATASET3	
	LogFC	FDR	LogFC	FDR	LogFC	FDR
*GBP4*	−2.45	2.34e−09	−2.52	1.72e−07	−2.07	1.63e−03
*IDO1*	−3.19	2.34e−09	−3.20	6.97e−09	−2.45	1.91e−04
*IGHV4-34*	3.55	2.80e−04	4.47	8.68e−08	3.63	2.48e−03
*LRP4*	−2.57	1.04e−05	−2.66	9.23e−05	–	–
*RNU6-196P*	3.60	4.69e−05	3.88	9.23e−05	–	–
*WIPF3*	−1.99	9.37e−05	−1.55	5.03e−04	–	–
*LGR6*	−1.91	3.76e−05	–	–	−2.03	8.07e−05
*CD8B*	−1.81	8.73e−05	–	–	−1.69	5-09e−03
*CD8A*	−1.62	2.08e−04	–	–	−2.13	4.20e−04
*APOL1*	−1.86	2.08e−04	–	–	−1.96	1.78e−03
*NELL2*	–	–	−2.76	9.23e−05	−3.09	1.78e−04
*CXCL11*	–	–	−3.06	1.00e−05	−3.01	1.78e−04

### Classification based on the DEGs

To classify primary patients that metastasize, five distinct classification methods were used: decision trees (DT), random forest (RF), linear and radial support vector machine (svmL and svmR, respectively), and logistic regression (LR). Due to high-dimensionality problems, the full gene expression dataset cannot be directly used. Therefore, we decide to perform feature selection using only DEGs found between early-stage patients that metastasize and those that do not metastasize. This is a common approach used to reduce feature dimension before classification. Since the number of DEGs found in each dataset was different, we used the 50 DEGs with the lowest *p*-value described above as means to use the same gene dataset dimension as input to all classifiers in all datasets tested. After training the classifiers 100 times, several performance evaluation metrics were calculated in the test set, such as accuracy, misclassifications, sensitivity, specificity, and area under the ROC curve (AUC). The median results of all runs obtained for each dataset in the test set are displayed on Table 4 (all performances for train and test sets may be found in the Additional file 1: Table S1). Also, pairwise comparisons of the accuracy obtained for classifiers may be found on Additional file 1: Table S2. It is shown that the results are similar between the different methods tested. Nonetheless, RF was the best classifier obtained, presenting higher accuracy (0.72, 0.71, and 0.71) and AUC (0.72, 0.71, and 0.69) in all datasets tested, and the lowest number of misclassifications (5) in the test set.

**Table 4:** Median classifiers performance results (and standard deviation in parenthesis) obtained for test sets for the 100 runs tested using five classification methods applied to the fifty DEGs with lowest *p*-value

	Acc	Miscl/FN	Sensitivity	Specificity	AUC
DT					
D1	0.61(0.098)	7(1.764)/3(1.465)	0.67(0.163)	0.56(0.167)	0.61(0.092)
D2	0.65(0.103)	6(1.746)/3(1.490)	0.63(0.186)	0.78(0.147)	0.65(0.102)
D3	0.59(0.101)	7(1.717)/3(1.371)	0.63(0.171)	0.67(0.176)	0.59(0.095)
$\bar{x}$	0.62	-	0.62	0.67	0.62
svmL					
D1	0.67(0.102)	6(1.844)/3(1.581)	0.67(0.176)	0.78(0.209)	0.67(0.102)
D2	0.71(0.092)	5(1.566)/3(1.589)	0.63(0.199)	0.78(0.152)	0.71(0.087)
D3	0.71(0.083)	5(1.415)/4(1.816)	0.50(0.227)	0.89(0.137)	0.69(0.088)
$\bar{x}$	0.70	–	0.60	0.82	0.69
svmR					
D1	0.67(0.101)	6(1.817)/3(1.662)	0.67(0.185)	0.56(0.199)	0.67(0.094)
D2	0.59(0.112)	7(1.909)/2(2.567)	0.75(0.321)	0.56(0.222)	0.61(0.114)
D3	0.53(0.090)	8(1.537)/6(1.798)	0.25(0.225)	0.89(0.221)	0.51(0.084)
$\bar{x}$	0.60	–	0.56	0.67	0.60
LR					
D1	0.67(0.092)	6(1.663)/3(1.282)	0.67(0.142)	0.67(0.163)	0.67(0.092)
D2	0.65(0.085)	6(1.441)/3(1.299)	0.63(0.162)	0.78(0.132)	0.64(0.082)
D3	0.65(0.105)	6(1.785)/3(1.428)	0.63(0.178)	0.72(0.188)	0.65(0.101)
$\bar{x}$	0.66	–	0.64	0.72	0.65
RF					
D1	0.72(0.089)	5(1.602)/3(1.132)	0.72(0.126)	0.78(0.140)	0.72(0.089)
D2	0.71(0.090)	5(1.524)/2(1.329)	0.75(0.166)	0.78(0.140)	0.71(0.091)
D3	0.71(0.102)	5(1.731)/4(1.450)	0.50(0.181)	0.89(0.158)	0.69(0.103)
$\bar{x}$	0.71	–	0.66	0.82	0.71

DT—decision trees; svmL—linear support vector machine; svmR—radial support vector machine; LR—logistic regression; RF—random forest; D1—DATASET1; D2—DATASET2; D3—DATASET3; $\bar{x}$—datasets mean; Acc—accuracy; Miscl—misclassifications; FN—false negatives; Sensitivity—fraction of actual positive cases (P); Specificity—fraction of actual negative cases (PM); AUC—area under the ROC curve

**Table 5:** Median values (and standard deviation in parenthesis) of the performance metrics in test set by regularized LR methods across 100 runs applied to the full dataset

	# Genes	Acc	Miscl/FN	Sensitivity	Specificity	AUC	# Common genes
EN							
D1	59(32.63)	0.67(0.093)	6(1.667)/3(1.133)	0.67(0.126)	0.67(0.142)	0.67(0.093)	8
D2	45(21.34)	0.59(0.102)	7(1.732)/5(1.387)	0.38(0.173)	0.78(0.166)	0.58(0.095)	4
D3	39(19.76)	0.59(0.074)	7(1.257)/6(1.135)	0.25(0.142)	0.89(0.118)	0.57(0.072)	6
$\bar{x}$	48	0.62	–	0.43	0.78	0.61	6
iTwiner							
D1	33(21.98)	0.78(0.075)	4(1.343)/4(1.362)	0.56(0.151)	1.00(0.036)	0.78(0.075)	19
D2	42(21.11)	0.65(0.056)	6(0.946)/6(0.904)	0.25(0.113)	1.00(0.040)	0.63(0.058)	25
D3	39(20.65)	0.65(0.050)	6(0.847)/6(0.783)	0.25(0.098)	1.00(0.040)	0.63(0.052)	30
$\bar{x}$	38	0.69	–	0.35	1	0.68	25

D1—DATASET1; D2—DATASET2; D3—DATASET3; $\bar{x}$—datasets mean; # Genes—number of genes selected by the methods; Acc—accuracy; Miscl—misclassifications; FN—false negatives; Sensitivity—fraction of actual positive cases (P); Specificity—fraction of actual negative cases (PM); AUC—area under the ROC curve; # Common genes—number of genes selected in common in at least 50% of the runs

### Regularized logistic regression

The second approach tested to distinguish early-stage CRC patients that metastasize from those that do not, was to use regularized LR with different types of penalization for feature selection: Elastic net (EN) and the correlation-based regularizer iTwiner. The test set results obtained for these methods applied to the full dataset are described in Table 5 (results for train and test sets may be found in the Additional file 1: Table S3).

The performance of these methods was similar to the classifiers tested above, where higher accuracy in test set was obtained by the iTwiner method (mean $\text{Acc} = 0.69$). Interestingly, most of the misclassifications in DATASET2 and DATASET3 using both approaches were false negatives (FN), meaning that these methods classified wrongly patients that do not metastasize in patients that metastasize. Since non-metastatic patients can indeed metastasize in the future it would be of great value to do a follow-up on these patients that were labeled wrongly by these methods.

**Fig. 4:**
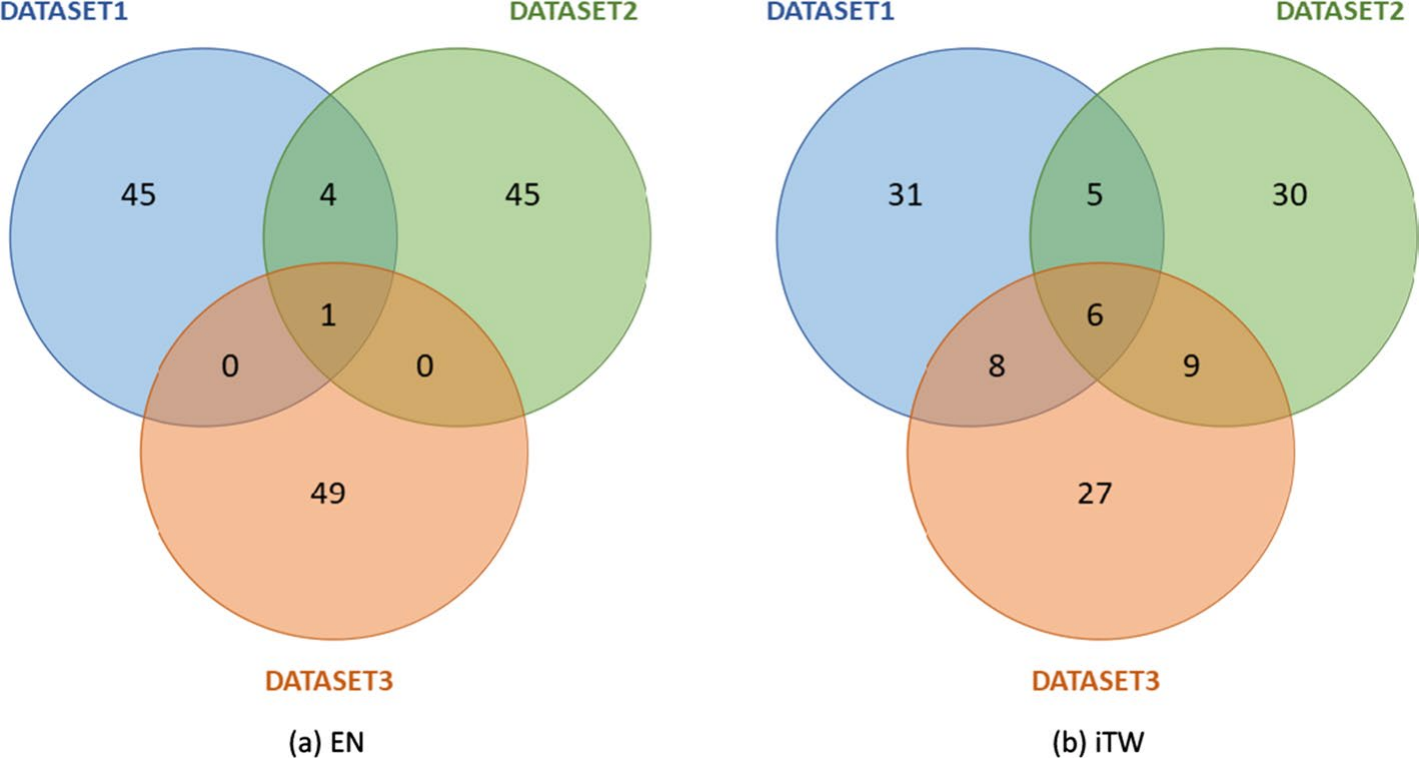
Venn’s diagram comparing the 50 genes that are selected more times by the regularization methods for each dataset tested. **a** Elastic net; **b** iTwiner

The median number of selected variables (genes) by the two methods (across the 100 runs) used to separate the two groups (P vs. PM) was 48 for EN and 38 for iTwiner (Table 5). Also, the number of genes selected in at least 50% of the 100 runs tested in EN was smaller when compared to iTwiner, indicating that the iTwiner method is more stable since more genes are consistently selected as important for the classification of early-stage patients that metastasize. Moreover, to assess which genes are being recurrently selected by the methods, independently of the dataset used, a Venn diagram was constructed (Fig. 4). Here, we compared for each regularizer (EN and iTwiner) the top fifty genes selected in each dataset (Table 6), as the most likely genes to be metastatic biomarkers in CRC patients. Interestingly, only a few biomarkers were found to be DEGs between the P and PM groups, represented in bold the down- and in underline the up-regulated genes in the PM group. Also, we can see that using iTwiner, a higher number of genes is selected in common across the three datasets tested, which once more stands as evidence of improved stability and robustness of the selected feature sets, irrespective of the dataset used.

Looking closely at the genes selected by each classifier in the different datasets tested (Table 6), EN only selected *RAC1P3* in common to all datasets. This gene is a pseudogene of the Rac family of small GTPase whose role in cancer is still unknown. Regarding iTwiner, six genes were selected by the three datasets tested, *RAC1P3, XRCC6P2, EEF1B2P6, HSPD1P7, TRBV11-1, HORMAD2*. The majority of these genes are pseudogenes with an unknown role in cancer. However, *HORMAD2* has been reported to have tumor suppressor functions, and its expression was seen down-regulated in cancer [50]. Here we showed that *HORMAD2* gene was down-regulated in early-stage patients that metastasize (represented in bold in Table 6).

**Table 6:** List of genes ranked by the number of times that were selected by regularized LR methods used. Genes colored in underline and bold represent DEGs found up- and down-regulate in PM tissues, respectively

**EN**						**iTwiner**					
**DATASET1**		**DATASET2**		**DATASET3**		**DATASET1**		**DATASET2**		**DATASET3**	
*PF4*	86	*RLN3*	86	*LRRC37A14P*	67	***RAC1P3***	100	*MIR602*	100	***NME2P2***	100
*RPL6P9*	81	*MIR602*	60	*MIR6829*	64	***XRCC6P2***	100	*RAC1P3*	100	*OPCML-IT2*	100
*LINC01871*	76	*NCOR1P4*	59	*MIR5002*	63	*OR51K1P*	98	***XRCC6P2***	100	***RAC1P3***	100
*CD8B*	69	*CD200*	56	*IGBP1P2*	56	*LINC01100*	96	*MIR3907*	96	***HORMAD2***	98
***XRCC6P2***	69	*ANTXRLP1*	38	*NMU*	54	*EEF1B2P6*	92	*KCTD9P3*	92	*LINC01100*	93
*SFRP5*	63	*OR1L4*	38	*MIR8078*	48	*HSPD1P7*	92	*GZMAP1*	88	***NMNAT1P3***	92
*SOHLH2*	60	*RPL6P9*	38	*MPPED1*	45	*MTCO2P18*	83	*TRBV11-1*	86	*MIR602*	88
*SLITRK1*	56	*VTI1BP4*	38	***NME2P2***	42	*TRBV11-1*	82	*LRIT1*	83	*NDUFA5P10*	84
*MIR1295A*	49	*RAC1P3*	36	*CNTN4-AS1*	39	*FABP7P2*	78	*PRSS57*	82	*KDM4F*	83
*TRAV41*	49	*RPL21P6*	35	*MIR658*	37	*HNRNPA1P42*	78	*RNU6-428P*	80	*TRBV11-1*	83
*TST*	48	*RCC2P1*	34	*ZNHIT1P1*	35	*KDM4F*	74	*LINC02543*	79	*APOOP4*	79
*NCOA4P2*	45	*TRAV14DV4*	34	*WDR82P2*	34	*IGKV2D-30*	69	*HAUS5-DT*	75	***IQCF5-AS1***	71
*BANK1*	42	*AHCYP2*	33	***HORMAD2***	33	*LINC01335*	69	*DNTT*	73	*MIR8078*	71
*ITM2A*	42	*NBPF13P*	33	*MIR659*	33	*RPL31P35*	63	*TPT1P3*	72	*H2BC2P*	68
*MGST1*	41	***XRCC6P2***	31	*MIR199B*	32	*SULT6B2P*	61	*OR51K1P*	71	*DNTT*	65
*LGR6*	40	*OR13J1*	30	***IGHV3-33***	31	*COX6CP7*	56	*HSPD1P7*	70	*IGHVIII-2-1*	64
*RNU6-1266P*	38	*RPL7AP53*	30	*TRDV3*	30	*LILRB1-AS1*	52	*RN7SKP32*	70	***KCTD9P3***	64
*DUOXA1*	37	*RPS2P35*	30	*HLA-DPA3*	29	*RPL7P58*	51	*OR1S2*	66	*TDGF1P7*	63
*LINC02866*	37	*MIR6851*	29	*TRAJ50*	29	*TRAJ20*	51	*RNU6-552P*	65	*OR1S2*	62
*FAM187A*	36	*MIR5088*	27	*TRDJ1*	29	*MIR376B*	50	*RPL32P17*	62	***VN2R9P***	62
*MYADM*	36	*PIN4P1*	27	*EEF1GP4*	28	*PPP1R14BP4*	49	*NDUFA5P10*	60	*MIR659*	61
*HSPD1P7*	34	*GOT2P1*	26	*MIR592*	25	*RNU6-1085P*	47	*CHMP1AP1*	58	*RPL7P12*	61
*PYCARD-AS1*	34	*BANK1*	25	*MIR6729*	25	*RPS27AP14*	47	*OR10H2*	58	*RPL21P104*	58
*CASQ1*	33	*WIPF3*	25	*RNA5SP396*	25	*LDHAP1*	45	*HORMAD2*	56	*LINC02059*	56
*IFNG*	33	*LINC01855*	24	*NPIPB6*	24	*PIN1P1*	43	*MIR564*	56	*EEF1B2P6*	55
*RNVU1-19*	33	*PPIAP54*	24	*SNORD31B*	24	*MTND3P18*	42	*RNU4-76P*	53	*TRAJ49*	54
*SMAD4*	33	*MIR7854*	23	*LINC02755*	23	*HORMAD2*	41	*SULT6B2P*	53	*RPL23AP26*	52
*BEND4*	31	*MIR877*	22	*MIR4725*	23	*MIR8052*	39	*EEF1B2P6*	50	*PPP1R14BP4*	51
*LINC00668*	31	*RPL26P9*	22	***RAC1P3***	23	*TRBJ2-1*	38	*MTND1P22*	50	*RNU6-973P*	48
*MIR6816*	31	*RBM11*	21	*RNU6-915P*	23	*TSHB*	38	*RNU1-18P*	49	*HSPE1P4*	47
*MPZL2*	31	*RNU6-28P*	21	*TRGV5*	23	*RPL23AP26*	37	*LINC00305*	48	*CHIAP3*	46
*BIK*	30	*RNU6-979P*	21	*LILRP2*	22	*RESP18*	36	*OR8G5*	47	*RNU6-179P*	44
*MPDU1*	30	*UBE2CP2*	21	*RNU6-33P*	22	*DNAJC19P3*	35	*SPANXN3*	46	***IGHV3-16***	42
*CDKL2*	27	*HEATR9*	20	*RNU6-875P*	22	*IL36A*	35	*TSHB*	46	*TLR12P*	42
***RAC1P3***	27	*LINC01845*	20	*LINC01290*	21	*MTND2P30*	35	*HPRT1P1*	43	*RPS27AP14*	40
*WIPF3*	26	*SNX19P2*	20	*MIR376B*	21	*HNRNPA3P14*	34	*TMPRSS11F*	43	*HNRNPCL1*	39
*C1DP5*	25	*TRBV7-4*	20	*NPM1P35*	20	*RNU6-337P*	33	*TRBJ1-6*	43	*MIR8052*	39
*DSC2*	25	*NPR2*	19	*RNA5-8SN1*	20	*FAM220BP*	32	*APOOP4*	41	***PRSS57***	39
*LINC01398*	25	*OR5K1*	19	*RNA5-8SN2*	20	*LINC02059*	32	*HNRNPA3P14*	39	*MRPL35P4*	38
*LTA*	25	*IBA57-DT*	18	*RNA5-8SN3*	20	*DNAJA1P6*	31	***MIR6508***	34	*DYTN*	37
*RNA5SP74*	25	*KCTD9P3*	18	***RPL9P18***	20	*LINC00951*	31	*LINC02868*	33	*MTHFD2P3*	36
*TTC30A*	25	*MT-TS2*	18	*MIR320C1*	19	*RNU6-948P*	31	*RPL31P28*	33	***XRCC6P2***	36
*LINC02734*	24	*OR7E62P*	18	*OR56A5*	19	*SMARCAL1-AS1*	31	*OR51B8P*	32	*HSPD1P7*	34
*MIR6776*	24	*CASP1P2*	17	*RNU6-1263P*	19	*DRD5P2*	29	*SCDP1*	31	*IQCF5*	34
*MIR7107*	24	*LINC00928*	17	*MIR5579*	18	*TDGF1P7*	28	*MIR6511A1*	30	***CHMP1AP1***	32
*SIT1*	24	*OOSP4A*	17	*RNU7-170P*	18	*TRAJ61*	28	*MIR6511A2*	30	*MIR6729*	32
*ENO1-AS1*	23	*CCDC40*	16	*RPS6P8*	18	*MIR6816*	26	*MIR6511A3*	30	*OR4K12P*	32
*ITGA6*	23	*KIR2DL4*	16	*SLAMF6P1*	18	*HAUS5-DT*	25	*MIR6511A4*	30	*RN7SKP242*	32
*AXIN2*	22	*SNORD13P1*	16	*KRT8P49*	17	*SLC25A5P9*	25	*OR1D3P*	30	*H2AZP5*	31
*FABP5*	22	*AKR1D1P1*	15	*SDR42E1P5*	17	*ALOX15P2*	24	*RNU6-917P*	30	*LRIT1*	31

### Classification based on regularized-selected genes

The final procedure to find a set of biomarkers involved in metastasis processes of early-stage CRC patients was to use classification methods based on the features previously identified by regularized LR. In particular, using the genes pre-selected by regularization (EN, iTwiner) as an alternative to the DEGs (Fig. 1, method 3), to try to improve the classification performance.

Here, the five classifiers used earlier (DT, svmL, svmR, LR, and RF) were applied to the two smaller gene sets obtained by regularized LR. To have the same dataset dimension as before, the fifty genes selected by EN and iTwiner penalties ranked in Table 6 were used as input to the classifiers. This was done to each dataset as previously. Performances obtained for classifiers train and test sets using genes pre-selected by EN and iTwiner penalties may be found on the Additional file 1: Tables S4 and S6, respectively. Pairwise comparisons of the accuracy obtained for classifiers may be found on Additional file 1: Tables S5 and S7.

Regarding classifiers applied to gene sets based on EN penalties (Table 7), for all datasets tested, the best results in test set were obtained using svmR ($Acc = 0.78, 0.76, 0.76$) and RF ($Acc = 0.78, 0.76, 0.76$) methods. Also, in the RF method, the specificity of the results was higher, i.e., most of the misclassifications were FN. This means that the classifier labeled patients as metastatic even though they were non-metastatic at the three years follow-up time.

Afterward, we tested the same classifiers applied to a different gene set based on iTwiner penalization (Table 8). The best accuracy was obtained by RF classifier as before ($Acc = 0.86, 0.82, 0.76$). However, using this iTwiner penalization improved the specificity of the classifier (Specificity = 1 for all datasets).

Table 9 presents the mean performance results for all the tested combinations of classifiers and feature selection methods: DEGs found between P and PM patient group (Table 9, DEG +), genes pre-selected by EN regularizer (Table 9, EN +) and genes pre-selected by the iTwiner (Table 9, iTwiner +). For all gene selection methods tested, the best performance classifier was RF showing the highest accuracy and specificity. Overall, using the genes found by regularization, considering different penalty vectors (and so different information used for selection), instead of using DEGs found between groups, improved in a significant way the accuracy of the classifiers (Table 9). A pairwise comparison using Wilcoxon rank sum test with Benjamini & Hochberg *p*-value correction was performed to assess the statistically significant differences between the groups (Additional file 1: Table S8).

**Fig. 5:**
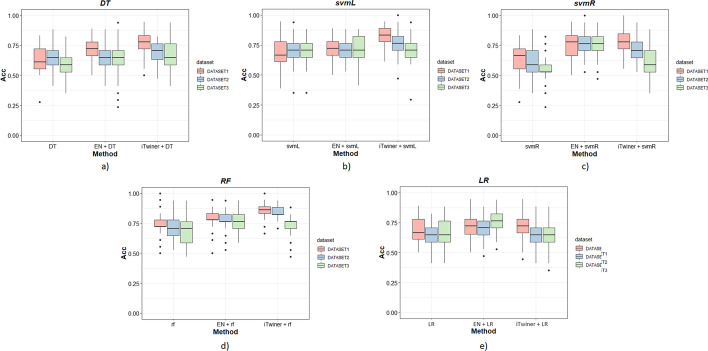
Boxplots comparing accuracy (Acc) obtained by the different approaches tested applied to each dataset. **a** Decision trees (DT); **b** linear support vector machine (svmL); **c** radial support vector machine (svmR); **d** random forest (RF); **e** logistic regression (LR)

Moreover, for most classifiers tested (DT, svmL, and RF), if the selection of genes is based on correlation matrices (iTwiner), the performance of the models increases significantly, leading to the most accurate results. To better visualize these, Fig. 5 shows boxplots of the classifiers’ accuracy obtained for all gene selection methods applied to each dataset tested. Overall, when gene sets were obtained by regularization (EN + and iTwiner +), higher accuracy was obtained. This is well observed in the RF classifier (Fig. 5d).

**Table 7:** Median values (and standard deviation in parenthesis) obtained in test set for the five classification methods using fifty most frequently genes pre-selected by LR with EN penalization

	Acc	Miscl/FN	Sensitivity	Specificity	AUC
DT					
D1	0.72(0.094)	5(1.698)/2(1.251)	0.78(0.139)	0.67(0.160)	0.72(0.094)
D2	0.65(0.093)	6(1.577)/3(1.282)	0.63(0.160)	0.67(0.168)	0.65(0.087)
D3	0.65(0.119)	6(2.021)/3(1.403)	0.63(0.175)	0.67(0.172)	0.65(0.098)
$\bar{x}$	0.67	–	0.68	0.67	0.67
svmL					
D1	0.72(0.081)	5(1.467)/4(1.439)	0.56(0.160)	0.89(0.118)	0.72(0.081)
D2	0.71(0.080)	5(1.355)/3(1.323)	0.63(0.165)	0.89(0.112)	0.70(0.082)
D3	0.71(0.093)	5(1.579)/3(1.463)	0.63(0.183)	0.78(0.130)	0.71(0.091)
$\bar{x}$	0.71	–	0.61	0.85	0.71
svmR					
D1	0.78(0.101)	4(1.812)/2(1.043)	0.78(0.116)	0.67(0.196)	0.78(0.101)
D2	0.76(0.095)	4(1.612)/1(0.964)	0.88(0.120)	0.67(0.184)	0.77(0.092)
D3	0.76(0.087)	4(1.480)/2(1.369)	0.75(0.171)	0.78(0.154)	0.76(0.088)
$\bar{x}$	0.77	–	0.80	0.71	0.77
LR					
D1	0.72(0.098)	5(1.757)/3(1.255)	0.67(0.139)	0.78(0.156)	0.72(0.098)
D2	0.71(0.092)	5(1.570)/3(1.331)	0.63(0.166)	0.78(0.146)	0.70(0.093)
D3	0.76(0.090)	4(1.535)/2(1.303)	0.75(0.163)	0.78(0.133)	0.76(0.091)
$\bar{x}$	0.73	–	0.68	0.78	0.73
RF					
D1	0.78(0.096)	4(1.722)/3(1.143)	0.67(0.127)	0.89(0.127)	0.78(0.096)
D2	0.76(0.078)	4(1.325)/2(1.037)	0.75(0.130)	0.89(0.107)	0.76(0.079)
D3	0.76(0.081)	4(1.384)/3(1.351)	0.63(0.169)	0.89(0.092)	0.76(0.085)
$\bar{x}$	0.77	–	0.68	0.89	0.77

DT—decision trees; svmL—linear support vector machine; svmR—radial support vector machine; LR—logistic regression; RF—random forest; D1—DATASET1; D2—DATASET2; D3—DATASET3; $\bar{x}$—datasets mean; Acc—accuracy; Miscl—misclassifications; FN—false negatives; Sensitivity—fraction of actual positive cases (P); Specificity—fraction of actual negative cases (PM); AUC—area under the ROC curve

**Table 8:** Median values (and standard deviation in parenthesis) obtained in test set for the five classification methods using fifty most frequently genes pre-selected by LR with iTwiner penalization

	Acc	Miscl/FN	Sensitivity	Specificity	AUC
DT					
D1	0.78(0.096)	4(1.721)/2(1.226)	0.78(0.136)	0.78(0.146)	0.78(0.096)
D2	0.71(0.085)	5(1.453)/3(1.344)	0.63(0.168)	0.78(0.143)	0.70(0.085)
D3	0.65(0.111)	6(1.882)/3(1.496)	0.63(0.187)	0.78(0.159)	0.65(0.108)
$\bar{x}$	0.71	–	0.68	0.78	0.71
svmL					
D1	0.83(0.071)	3(1.284)/3(1.288)	0.67(0.143)	1.00(0.022)	0.83(0.071)
D2	0.76(0.089)	4(1.152)/3(1.218)	0.63(0.152)	1.00(0.085)	0.75(0.092)
D3	0.71(0.092)	5(1.568)/4(1.256)	0.50(0.157)	0.78(0.130)	0.69(0.084)
$\bar{x}$	0.77	–	0.60	0.93	0.76
svmR					
D1	0.78(0.089)	4(1.602)/2(1.015)	0.78(0.113)	0.78(0.153)	0.78(0.089)
D2	0.71(0.097)	5(1.656)/2(1.326)	0.88(0.120)	0.78(0.188)	0.72(0.096)
D3	0.59(0.109)	7(1.487)/4(2.259)	0.50(0.282)	0.78(0.154)	0.58(0.107)
$\bar{x}$	0.69	–	0.72	0.78	0.69
LR					
D1	0.72(0.094)	5(1.687)/3(1.431)	0.67(0.159)	0.78(0.130)	0.72(0.094)
D2	0.65(0.097)	6(1.652)/4(1.406)	0.50(0.176)	0.89(0.151)	0.64(0.097)
D3	0.65(0.096)	6(1.633)/4(1.456)	0.50(0.182)	0.67(0.173)	0.63(0.092)
$\bar{x}$	0.67	–	0.56	0.78	0.66
RF					
D1	0.86(0.063)	3(1.132)/2(1.104)	0.78(0.123)	1.00(0.025)	0.86(0.063)
D2	0.82(0.058)	3(0.983)/3(1.003)	0.63(0.125)	1.00(0.040)	0.81(0.061)
D3	0.76(0.075)	4(1.267)/4(1.135)	0.50(0.142)	1.00(0.102)	0.75(0.076)
$\bar{x}$	0.81	–	0.64	1	0.81

DT—decision trees; svmL—linear support vector machine; svmR—radial support vector machine; LR—logistic regression; RF—random forest; D1—DATASET1; D2—DATASET2; D3—DATASET3; $\bar{x}$—datasets mean; Acc—accuracy; Miscl—misclassifications; FN—false negatives; Sensitivity—fraction of actual positive cases (P); Specificity—fraction of actual negative cases (PM); AUC—area under the ROC curve

**Table 9:** Mean performance metrics values of the three datasets tested (test set) obtained for the classification methods applied to different gene sets based on DEGs, EN and iTwiner

Combined methods	Acc	Sensitivity	Specificity	AUC
DEGs+				
*DT*	0.62	0.62	0.67	0.62
*svmL*	0.70	0.60	0.82	0.69
*svmR*	0.60	0.56	0.67	0.60
*LR*	0.66	0.64	0.72	0.65
*RF*	0.71	0.66	0.82	0.71
EN +				
*DT*	0.67	0.68	0.67	0.67
*svmL*	0.71	0.61	0.85	0.71
*svmR*	0.77	0.80	0.71	0.77
*LR*	0.73	0.68	0.78	0.73
*RF*	0.77	0.68	0.89	0.77
iTwiner +				
*DT*	0.71	0.68	0.78	0.71
*svmL*	0.77	0.60	0.93	0.76
*svmR*	0.69	0.72	0.78	0.69
*LR*	0.67	0.56	0.78	0.66
*RF*	0.81	0.64	1.00	0.81

Acc—accuracy, Sensitivity—fraction of actual positive cases (P); Specificity—fraction of actual negative cases (PM), AUC—area under the ROC curve

## Conclusions

CRC is one of the leading causes of cancer-related deaths worldwide, being metastasis the major cause in these patients. Therefore, it is crucial to accurately diagnose CRC at an early-stage and understand the molecular mechanisms underlying metastasis. Several studies have tried to understand tumor biology and metastasis mechanisms by comparing early-stage versus metastatic tumors. We explore the relevance of studying early-stage (II-III) tumors that do not metastasize versus those that metastasize, in three years of follow-up. However, this is not an easy task since the high-dimensionality of gene expression data leads to problems in classification methods. As such, feature selection methods are important for selecting informative genes prior to classification, to improve their accuracy.

Here we present two major contributions to the discovery of metastatic biomarkers in CRC based on classification and feature selection. The first contribution is a new network-based feature selection method, iTwiner, that promotes the selection of genes with distinct correlation patterns in metastatic and non-metastatic patients, and has shown to significantly increase the classifiers’ predictive performance. Moreover, the proposed iTwiner regularizer selected the most stable and robust gene sets, including tumor suppressor genes and genes involved in several cancer processes like tumor growth and metastasis.

The second contribution proposes using gene sets pre-selected by regularized LR (via EN and iTwiner) as input features in the classification learning task, with proven improved performance compared to using DEGs as features, across many datasets and classifiers tested. Correlation-based penalization via the iTwiner penalty selected the best gene set for accurately distinguishing the two groups of patients, placing iTwiner as a promising strategy in the classification of CRC patients based on RNA-seq data and for the disclosure of biomarkers of CRC metastasis.

As future work, other types of classifiers may be tested, such as Gradient Boosting, Gaussian Process or neural networks, and since different hyper-parameter values may affect the classifiers’ performance, a more in depth investigation on optimization and tuning of parameters should be addressed. Also, studying the output of the binary classifiers and comparing those with genes selected by regularization methods would be an interesting next step, followed by gene function analysis to describe the biological role of genes and find potential enriched mechanisms and pathways.

## Supplementary Information

**Additional file 1**. Performance results and comparison of classifiers.

## Data Availability

Two cohorts of CRC patients from Hospital Santa Maria (Lisbon, Portugal): Cohort 1: Cohort described in [27] containing 111 samples, already available under accession number EGAS00001005276 (European Genome-Phenome Archive) - https://ega-archive.org/search-results.php?query=EGAS00001005276; Cohort 2: Cohort described in [28] containing 114 samples, already available in NCBI Database under accession number PRJNA689313 - https://www.ncbi.nlm.nih.gov/bioproject/PRJNA689313. Code used is available at https://github.com/sysbiomed/iTwiner.git.

## Abbreviations

CRC: Colorectal cancer
RNA-seq: RNA sequencing
DEGs: Differentially Expressed Genes
FAP: Familial Adenomatous Polyposis
NGS: Next-Generation Sequencing
LR: Logistic Regression
DTs: Decision Trees
RF: Random Forest
SVMs: Support Vector Machines
ESMO: European Society for Medical Oncology
NCCN: National Comprehensive Cancer Network
AJCC: The American Joint Committee on Cancer
WTS: Whole Transcriptome Sequencing
P: Early stage patients that do not metastasize
PM: Early stage patients that metastasize
FDR: False discovery rate
svmL: Linear support vector machine
svmR: Radial support vector machine
EN: Elastic net
TP: True Positive
FP: False Positive
TN: True Negative
FN: False Negative
Acc: Accuracy
Miscl: Misclassification
AUC: Area Under the Curve
ROC: Receiver Operator Characteristic curve
logFC: Log Fold Change
